# Training imaging professionals on transgender-inclusive breast cancer screening practices

**DOI:** 10.1007/s10552-026-02241-1

**Published:** 2026-09-18

**Authors:** Arielle Martinez, Sarah Whitton, Jonah Yokoyama, Jules Madzia, Carson S. Hartlage, Elizabeth Kelly, Madeline Schumacher, Delia Sosa, Harsimran Makkad, Catherine Xu, Rahul Patel, Sarah Pickle, Shanna Stryker

**Affiliations:** 1 University of Cincinnati College of Medicine, Cincinnati, USA; 2 Department of Psychology, University of Cincinnati College of Arts & Sciences , Cincinnati, USA; 3 Equitas Health, Columbus, USA; 4 Department of Sociology, University of Cincinnati College of Arts & Sciences, Cincinnati, USA; 5 Department of Family and Community Medicine, University of Cincinnati College of Medicine, Cincinnati, USA; 6 Department of Psychiatry & Behavioral Neurosciences, University of Cincinnati College of Medicine, Cincinnati, USA

**Keywords:** Transgender, Breast cancer screening, Education, Mammography

## Abstract

**Purpose:**

Transgender/gender diverse (TGD) people are underscreened for breast cancer. A barrier to screening is anticipated discrimination during screening encounters. This pilot study evaluates a training session for breast imaging professionals (clinical support specialists, technologists, and radiologists) on LGBTQIA+-inclusive breast cancer screening visits.

**Methods:**

Training topics included terminology, an overview of the guidelines, principles of trauma-informed care, and strategies for creating a welcoming and inclusive environment. Pre- and post-surveys gathered participants’ demographic information, frequency of LGBTQIA+ patient interactions, and previous exposure to LGBTQIA+ training and assessed changes in knowledge, self-reported knowledge, and attitudes toward gender diversity.

**Results:**

In pre-surveys, participants reported frequent interactions with LGBTQIA+ patients and little previous training. A 45-min didactic training that included storytelling of relevant experiences resulted in a significant increase in participants’ self-reported knowledge of LGBTQIA+ terminology with a large effect size. After training, participants correctly answered more directly assessed knowledge questions (small effect size), with a trend toward attitudes more accepting of gender diversity (medium effect size), but neither reached statistical significance.

**Conclusion:**

Continued efforts to implement and evaluate inclusive education for the entire clinical team are essential to enabling more inclusive and welcoming clinical encounters that will reduce the disparities in breast cancer screening for TGD populations.

**Supplementary Information:**

The online version contains supplementary material available at https://doi.org/10.1007/s10552-026-02241-1.

## Background

Nearly 1 in 8 women in the U.S. will get breast cancer, which is the second-leading cause of cancer death in cisgender women [1]. Early detection is critical for decreasing mortality from breast cancer and is accomplished with screening using clinical breast examination, mammogram, molecular breast imaging, ultrasound, and/or magnetic resonance imaging.

Lesbian, gay, bisexual, transgender, queer, intersex, asexual, and other gender/sexual minority (LGBTQIA +) individuals, particularly transgender/gender diverse (TGD) individuals, have medication-related, behavioral, and reproductive risk factors for breast cancer (hereafter abbreviated BCC for ‘breast/chest cancer’ as a gender-inclusive term to refer to cancer which originates at the anatomic breast tissue; we intend this term as non-inclusive of thoracic cancers of the chest wall or of tissues or organs inside of the chest) [2]. While many reports of BCC in LGBTQIA+ individuals have been documented, the true prevalence remains unclear due to inconsistent and inaccurate collection of sexual orientation and gender identity data in clinical and cancer registries; data that are available show delays in BCC diagnosis and a 3-fold higher chance of recurrence [2, 3]. Contributing to these delays, LGBTQIA+ individuals, particularly TGD individuals, are underscreened for BCC due in part to low awareness about TGD BCC screening guidelines among primary care clinicians and gynecologists and, importantly, also due to anticipated discrimination [4–11].

Our study aims to evaluate the efficacy of a novel didactic training for imaging professionals on LGBTQIA+-inclusive screening encounters in improving participants' knowledge and attitudes toward gender diversity. Ultimately, such efforts could improve the BCC screening experience and thereby increase screening rates and lower BCC mortality.

## Activity

### The training program

A 45-min virtual training session was developed by students from the University of Cincinnati (UC) College of Medicine and the Gender-Affirming Care Clinical Specialist at Equitas Health, a community health center dedicated to serving the LGBTQIA+ community. The content was adapted from other cultural responsiveness trainings designed and led by the Gender-Affirming Care Clinical Specialist, a nurse with over a decade of experience training hundreds of medical professionals over the last decade and adapted to include content salient to an audience that included clinical service specialists involved in scheduling breast cancer screening imaging, radiology technologists, and radiologists. Most members of the team who developed the training content were inspired by their own lived experience as LGBTQIA+ community members, several of whom identified as TGD individuals. Content included terminology pertinent to the LGBTQIA+ community, the importance of inclusive communication and screening practices, an overview of current TGD BCC screening guidelines, and an emphasis on strategies to make the clinical space more inclusive for TGD patients. Specific content on respectful and inclusive care of TGD individuals was emphasized given the unique needs of the TGD community even within the larger LGBTQIA+ community related to BCC screening [12]. The presentation was delivered virtually to breast center team members at a large urban academic health center, including community-based team members working on mobile mammography units. The training used a slide deck and incorporated storytelling of relevant lived experiences. The presentation was offered over the lunch hour, with participants joining from a location that was convenient for them and leadership confirming that this was paid time in which there was not patient care duties; continuing education credit was offered upon completion, but participation in our evaluation surveys was not required for continuing education credit.

### Evaluation

In this study, participants were given a QR code and link to complete an electronic survey via Research Electronic Data Capture (REDCap) immediately before (pre) and after (post) the training on their personal devices; participation was voluntary. This study was intended to improve the experiences of patients at our institution and was determined not to meet the criteria for human subjects research by the UC Institutional Review Board.

In the pre-intervention survey, participants reported cultural background, gender identity, and relationship to the LGBTQIA+ community (self-identity, family members, close friends, or none of the above). Participants also described their job title, the frequency with which they interacted with lesbian, gay, and bisexual patients, with transgender patients, with nonbinary patients, and if they had prior training in LGBTQIA+ patient care and felt that they had access to sufficient materials on LGBTQIA+ care. Participants were invited to share more information about why and how they would like more training in an open-response field.

Knowledge was assessed using two methods. First, participants were asked to self-rate their level of knowledge of five key learning objectives (definition of LGBTQIA+; the use of LGBTQIA+-inclusive terminology in a medical setting; current BCC guidelines and recommendations for LGBTQIA+ patients; ways to create a welcoming environment for LGBTQIA+ patients; and the difference between sex, gender, gender expression, and sexual orientation) using a four-point Likert scale (1 = Not knowledgeable; 4 = Very knowledgeable). Ratings were averaged across items (Cronbach’s alpha = 0.90 at pre, 0.91 at post).

Second, knowledge was evaluated with a series of eight multiple-choice questions that directly assessed the content covered in the training, including scenario-based questions where participants were asked to pick the best option(s) to respond to the situation. These questions were pilot-tested with five individuals prior to the study to optimize readability, flow, and a reasonable degree of difficulty that would be able to detect increases in relevant knowledge. They were developed based on the professional and lived experience of several team members, including the Gender-Affirming Clinical Care Specialist. Scenarios included calling a TGD patient from the waiting room, correcting a verbal pronoun mistake, and confirming a patient’s identity when the listed appointment name does not match the listed legal name. The remaining questions asked the participants to identify features of a welcoming environment and to describe a key feature of trauma-informed care covered in the training. For analyses, participant responses to each question were coded as correct (1) or incorrect (0); we summed these values across items to indicate the total number of items answered correctly (possible values from 0–12).

Finally, attitudes toward gender diversity were assessed using an adaptation of a transphobia scale developed by Stroumsa et al. in 2019 [13]. Participants were asked to rate how much they agreed with eight statements about gender (e.g., “It is important for me to be able to identify someone as only a man or only a woman upon meeting them”) on a 7-point Likert scale (1 = Strongly Agree; 7 = Strongly Disagree). Ratings were averaged across items (Cronbach’s alpha = 0.85 at pre, 0.91 at post); higher scores reflect more accepting attitudes toward gender diversity.

### Analytic plan

Descriptive statistics were calculated to describe participants’ demographics and experience. Paired-samples *t*-tests were used to assess within-person changes from pre- to post-intervention on key outcomes (self-reported knowledge, directly assessed knowledge, and attitudes toward gender diversity). Because this was a pilot study with a small sample, we focused on estimating effect sizes for pre-to-post changes to inform future larger-scale evaluations [14]. Effect sizes were estimated using Hedges’ g, which corrects for small sample size. Nevertheless, we also calculated statistical significance of pre-to-post changes.

## Results

### Participants

There were 28 attendees, with 17 participants completing the pre-survey (61%) and 14 completing the post-survey (50%). Participant demographics are summarized in Table 1. Slightly over half (*n*=8, 53%) of our cohort were mammogram technologists; the others were ultrasound technologists, clinical support staff, radiologists, or managers.

**Table 1:**
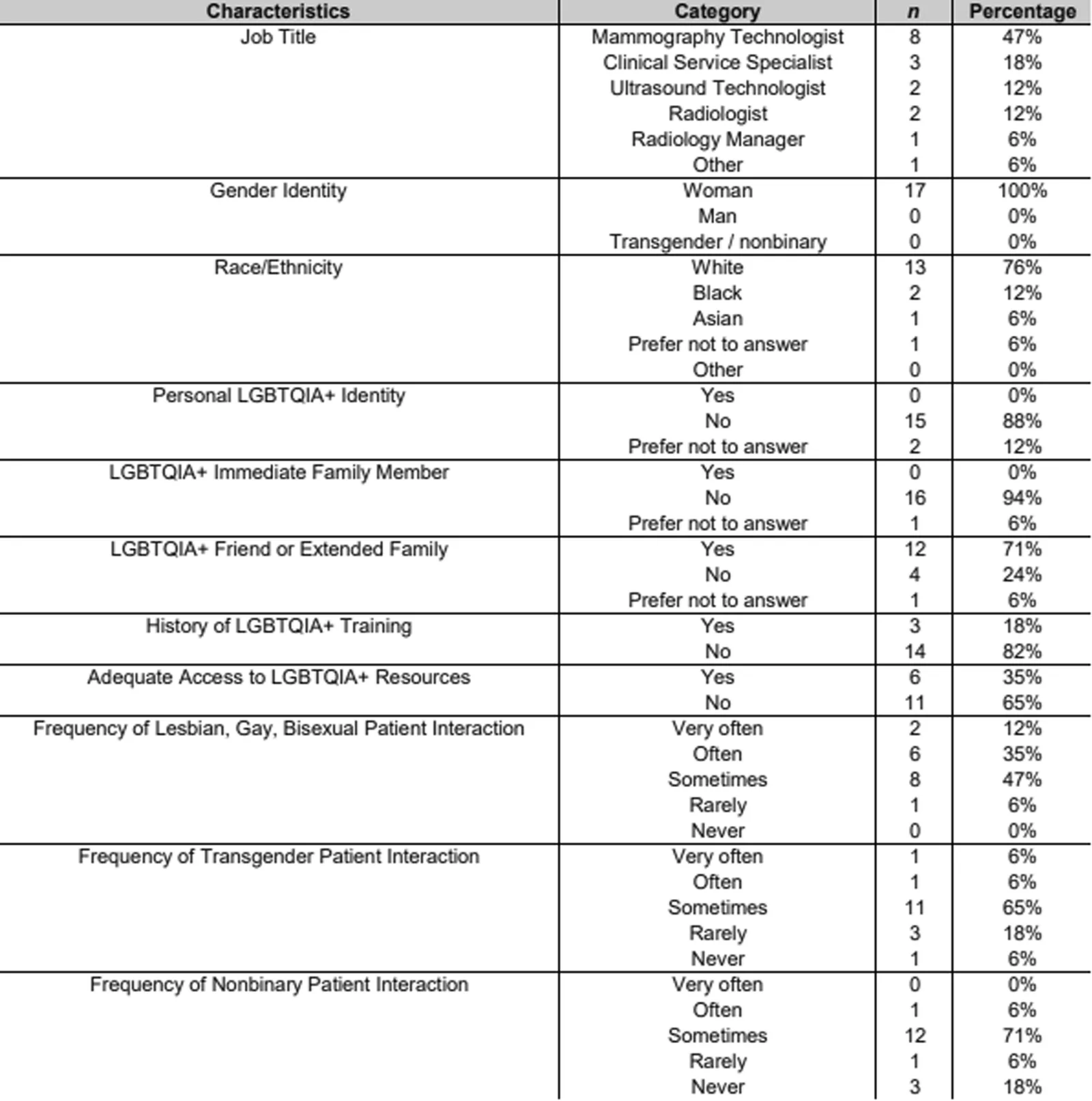
Demographics of participants

LGBTQIA+: lesbian, gay, bisexual, transgender, queer, intersex, asexual, and other gender/sexual minority

### Relevance of training

The relevance of this training was confirmed by our pre-intervention data showing that most (*n*=13, 77%) of the participants reported sometimes, often, or very often working with TGD patients and almost all (*n*=16, 94%) reported sometimes, often, or very often working with LGB patients. Despite this, only three participants had received LGBTQIA+-specific training (both radiologists and one mammography technologist), and only a third of participants (*n*=6, 35.3%) felt they had adequate access to resources about LGBTQIA+ patients. In open-field responses, they described barriers to learning including discomfort about discussing this topic among others, not knowing where to start, and being unsure of how to support LGBTQIA+ patients without unintentionally singling them out.

### Tests of program effects

**Fig. 1:**
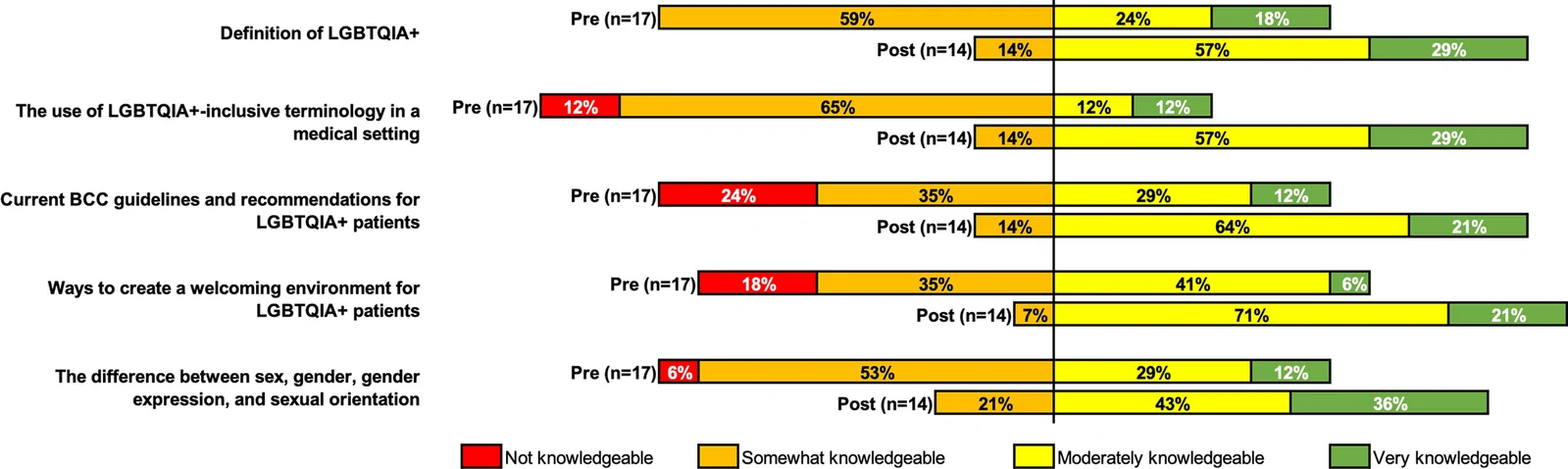
Self-reported knowledge pre- and post-intervention. LGBTQIA+: lesbian, gay, bisexual, transgender, queer, intersex, asexual, and other gender/sexual minority

**Fig. 2:**
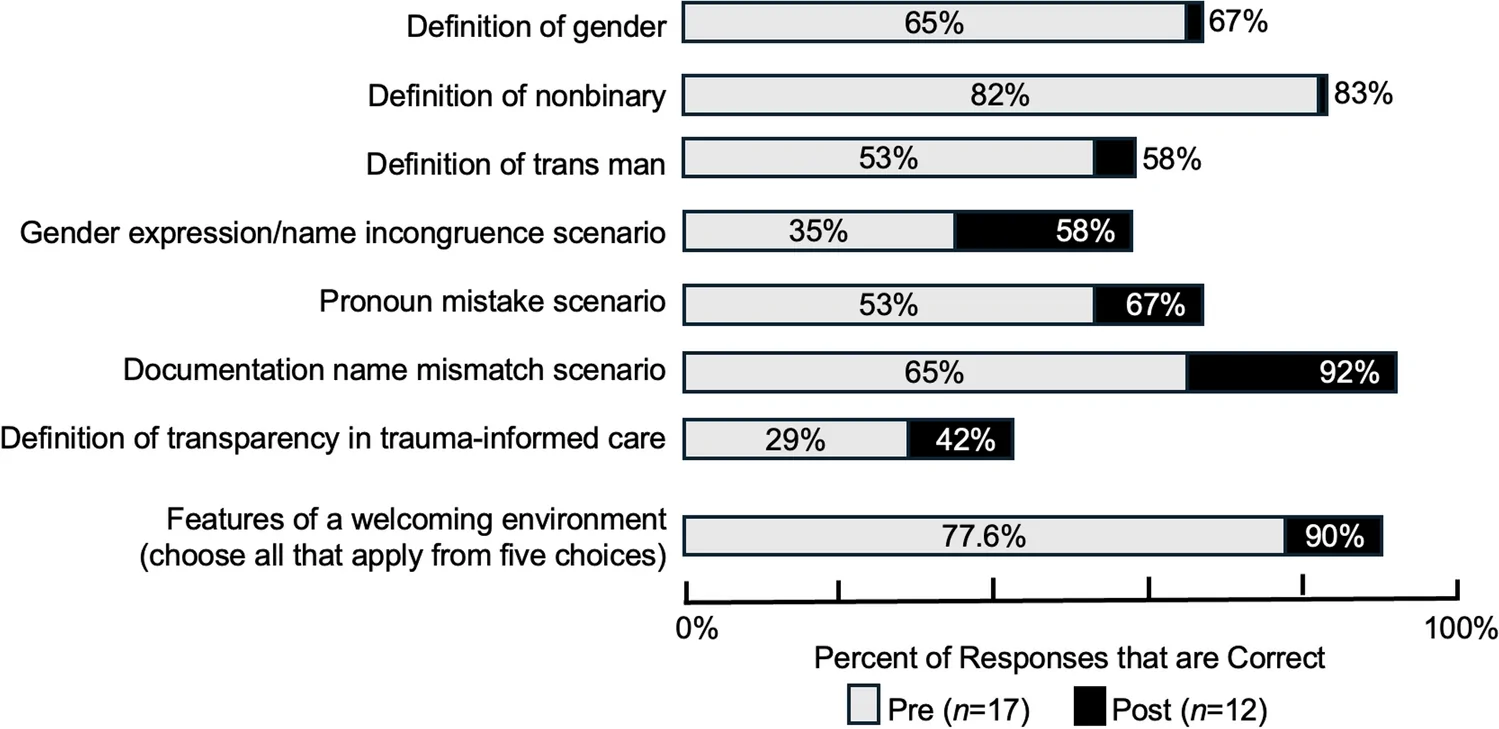
Directly assessed knowledge

**Fig. 3:**
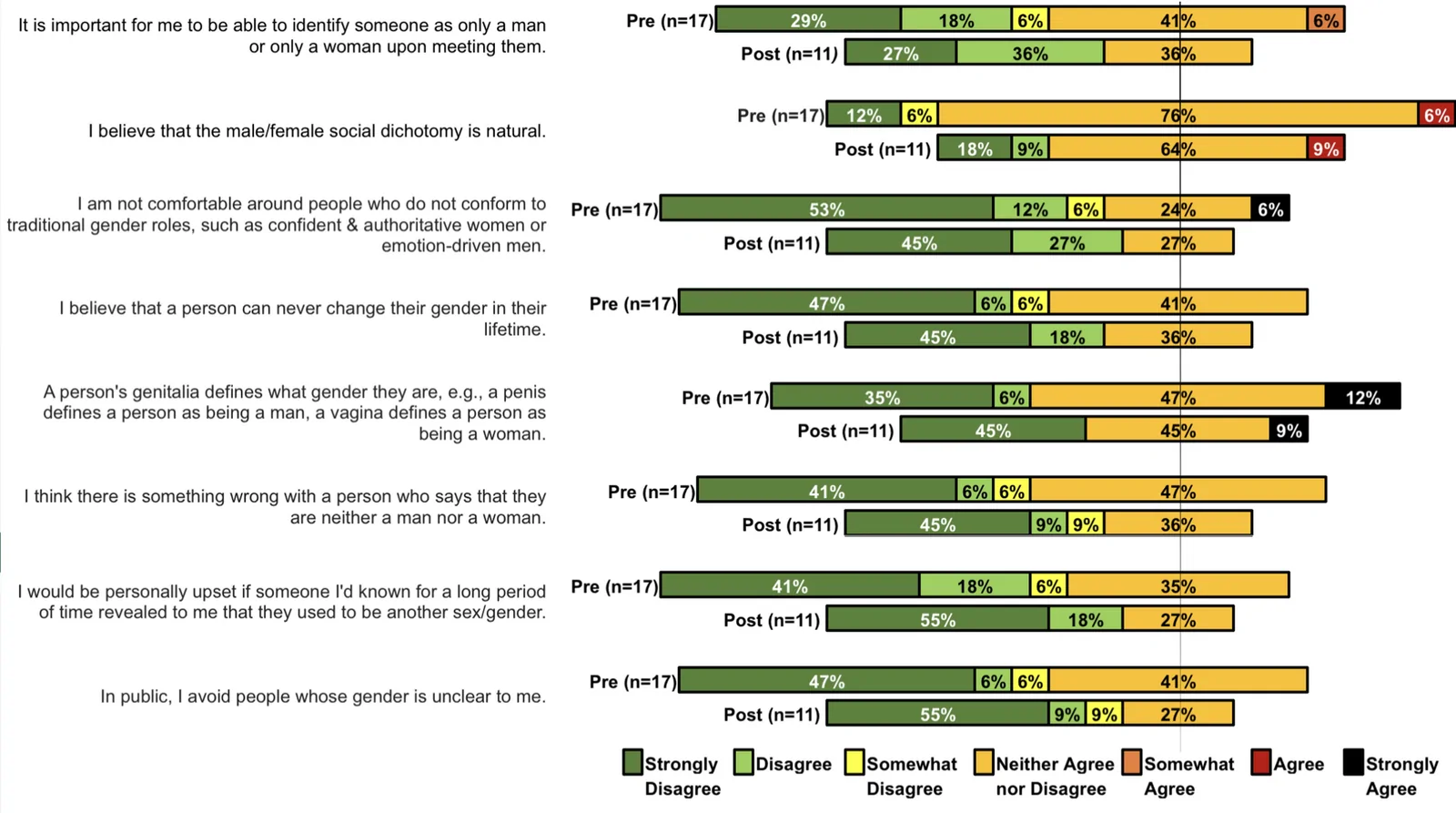
Gender attitudes scale scores

Paired-samples t-tests revealed that self-reported knowledge increased from pre-intervention, *Mean* = 2.4 (SD = 0.75), to post-intervention, *Mean* = 3.13 (SD = 0.56), t = 4.39; *p* = 0.001. Hedges’ g = 1.1, indicating a large effect size. We also estimated pre- to post- changes on each of the five knowledge items separately, as seen in Fig. 1; self-reported level of knowledge on each of the five individual items increased at a statistically significant level (all *p*s <0.02). As seen in Fig. 2, the number of multiple-choice knowledge questions answered correctly increased slightly from pre-intervention, *Mean* = 8.08 (SD = 1.73), to post-intervention, *Mean* = 9.16 (SD = 2.12), with a small effect size (Hedges’ *g* = 0.32). However, this increase was not statistically significant, *t* = 1.18; *p* = 0.26. As seen in Fig. 3, Gender Attitudes Scale scores also increased from pre-intervention, *Mean* = 42.36 (SD = 9.15), to post-intervention, *Mean* = 44.36 (SD = 10.25), with a medium effect size (Hedges’ *g* = 0.48), indicating a shift toward greater acceptance of gender diversity. However, this increase was not statistically significant, *t* = 1.71; *p* = 0.12. The full statistical output for these analyses is included in supplemental materials.

## Discussion

Our findings confirm that for many breast center team members, training opportunities in LGBTQIA+ care are rare despite frequent interactions with LGBTQIA+ patients. After a 45-min training, imaging professionals’ self-reported knowledge of the LGBTQIA+ community improved significantly. To our knowledge, this is the first published training aimed at the entire team of imaging professionals involved with BCC screening. Our team has previously received positive qualitative feedback from radiology residents on a transgender curriculum (not focused on BCC), but did not measure changes to knowledge [15].Clinical vignettes demonstrating inclusive terminology and evidence-based recommendations for TGD patients seeking breast imaging have been published to address a knowledge gap among physicians, and the authors emphasizes the need to educate all members of the imaging team [16]. To our knowledge, our work is the first that highlights the impact of education for all critical patient-facing team members, including technologists and clinical service specialists.

The improved self-reported understanding of the LGBTQIA+ community reported by our participants aligns with the findings from a study of interprofessional emergency department team members and with a review of brief training sessions for healthcare providers [17, 18]. Our training did not show improvement in gender attitudes and directly assessed knowledge, unlike other published studies with a similar training structure but larger and different participant groups [19, 20]. It is possible that we did not demonstrate statistically significant improvements in knowledge due to our smaller sample size.

In addition to sample size, another major limitation was our retention rate, which we attributed to insufficient time allocated for the surveys within the one-hour time slot due to participants needing to prepare to start patient care after the lunch hour. Important next steps include evaluating the training’s impact on patients, such as measuring changes in anticipated discrimination in patients who have experienced affirming BCC screening encounters. Participant feedback highlighted that the most valued component was storytelling of trainers’ lived experiences; therefore, incorporating additional narratives may strengthen the connection to the material. With these future steps, we aim to advance LGBTQIA+ cultural humility in cancer screening spaces and ultimately decrease BCC mortality.

## Supplementary Information

Below is the link to the electronic supplementary material.

Supplementary file1 (PDF 95 KB)

Supplementary file2 (PDF 523 KB)

## Data Availability

The datasets generated and analyzed during the current study are not publicly available due to concerns for privacy but are available from the corresponding author on reasonable.
