# Supplementary material for: Training imaging professionals on transgender-inclusive breast cancer screening practices
Source: Cancer Causes Control. 2026 Sep 18;37(10):163. doi: 10.1007/s10552-026-02241-1 (PMC13588859; doi:10.1007/s10552-026-02241-1)
Supplement: Supplementary file 1 [file 10552_2026_2241_MOESM1_ESM.pdf]

PRE-SURVEY

Exposure to LGBTQIA+ Health Education

- What is your role/job title?
  - Clinical Service Specialist
  - Mammography technologist
  - Ultrasound technician
  - Radiologist
  - Radiology Manager
  - Other
- What year did you start working in your current position (at any organization)? Please enter a four-digit year.
  - (1950-present)
- Do you work on (or schedule for) the mobile mammography van at UC Health?
  - Often
  - Sometimes
  - Rarely
  - Never
- Have you ever attended training on how to serve the LGBTQIA+ community?
  - Yes
    - If yes, how many hours of training have you completed? Please enter a numerical value.
  - No
- Do you feel like you have adequate access to resources and materials about LGBTQIA+ patients?
  - Yes
    - If yes, what resources do you use?
  - No
    - If no, what barriers have you observed in receiving this access?
- At work, how often do you estimate you have interacted with patients from the following groups (very often, often, sometimes, rarely, never):
  - Lesbian/Gay/Bisexual/Queer
  - Transgender
  - Gender non-binary / genderqueer

Self-Assessed Knowledge Scale

Please rate your knowledge on the following BEFORE completion of the training:

	**Very knowledgeable**	**Knowledgeable**	**Aware but do not know much about it**	**Not at all knowledgeable**
Definition of LGBTQIA+				
Difference between sex, gender identity, gender expression, and sexual orientation				
Use of LGBTQIA+-inclusive terminology in medical setting				
Current breast cancer guidelines & recommendations for LGBTQIA+ patients				
Ways to practice demonstrating support and a safe place for LGBTQIA+ patients				

Knowledge Survey

1. Out of the options provided, what best represents the definition of gender?
   - A person's internal self-understanding and self-identification
     - Correct choice
   - The way a person presents themselves to the world
   - The expression of genetic information in the body
   - A person's genetic makeup (XX, XY chromosomes)

2. Which of these describes a person with a non-binary gender identity?
   - Someone who is attracted to both men and women
   - Someone who is attracted to neither men or women

- Someone who identifies as a woman but was called a boy when they were born
- Someone who identifies as neither a man nor a woman
    - Correct choice

3. Which of these describes a trans man?
    - A man who is attracted to other men
    - A man who is attracted to men and women
    - A person who was designed male at birth but identifies as a woman
    - A person who was designated female at birth but identifies as a man
        - Correct choice
    - A person who was designated male at birth but identifies as a man sometimes and a woman other times
    - A person who was designated female at birth and identifies as neither a man nor a woman

4. Which of these are environmental features of healthcare spaces that can make a transgender or non-binary person feel welcome? (choose all that apply)
    a. Having separate male and female bathrooms
    b. Staff wearing pronoun pins/stickers
    c. Gender-neutral names for spaces (e.g. “Center for Reproductive Health” rather than “Center for Women’s Health”)
    d. Intake forms that allow someone to choose between “male” “female” and “other” for sex
    e. Intake forms that allow for someone to indicate their chosen or preferred name
        i. Correct answers b, c, e

5. Which would be the most inclusive way of calling someone named “John Smith” who was wearing a dress and had long hair from the waiting room?
    - (at door) “Mrs. Smith?” (behind door) “Hello, I’m Taylor and I use she/her pronouns. Let’s go back to the room”
    - (at door) “Mr. Smith?” (behind door) “Hello, I’m Taylor. Let’s go back to the room”
    - (at door) “Smith?” (behind door) “Hello, can you confirm your date of birth? I’m Taylor and I use she/her pronouns. Let’s go back to the room”
        - Correct choice
    - (at door) “Smith?” (behind door) “Hello, I’m Taylor. Let’s go back to the room”

6. You are the clinical service specialist and ask the partner of patient Jane (legal name John) Smith who uses she/her pronouns what their relationship to “him” is. The partner corrects you. What is the most appropriate next thing to say?
    - “I’m so sorry, I’m new to this. I’ll do better next time.”
    - “I’m sorry, it’s so confusing when it says John on here. I’m trying to learn to get better at this.”
    - “I’m sorry, what’s your relationship to *her*? Are you her emergency contact? Can I have her insurance card?”

- Correct choice

7. You are the clinical service specialist and a person with a beard comes to your station in the waiting room to check in for a mammogram. You ask for his name and he says "Matthew Jones". You do not see an appointment today for a Matthew Jones, but see one in a few minutes for a "Jane Jones". What would be the best response?
    a. "Are you transgender? I see a different name in here" Then update legal name in the computer.
    b. "Can you confirm your date of birth and address?" It if matches date of birth and address for Jane Jones, tell him that he is registered under Jane and will need to give that name next time to confirm identity.
    c. "Can you confirm your date of birth and address?" It if matches date of birth and address for Jane Jones, ask for an ID. If it says Jane, add "Matthew" as chosen name and/or tell staff so they know to call him Matthew.
        i. Correct choice
    d. "Could it be under Jane Jones?" Then update chosen name in the system to Matthew

8. What is an example of transparency in Trauma-Informed Care?
    a. Allowing patients to take breaks or stop an exam/procedure if needed
    b. Explaining the importance of each step of a procedure and what to expect
        i. Correct choice
    c. Only asking questions relevant to your role on the team (e.g. not asking about genital surgery if you do not need to know)
    d. Use proper draping techniques or ensuring privacy before asking sensitive questions

Gender Attitudes Scale

(all scored on a seven-point Likert scale from Strongly Agree to Strongly Disagree)

1. It is important for me to be able to identify someone as only a man or only a woman upon meeting them.
2. I believe that the male/female social dichotomy is natural.
3. I am not comfortable around people who do not conform to traditional gender roles, such as confident & authoritative women or emotion-driven men.
4. I believe that a person can never change their gender in their lifetime.
5. A person's genitalia defines what gender they are, e.g., a penis defines a person as being a man, a vagina defines a person as being a woman.
6. I think there is something wrong with a person who says that they are neither a man nor a woman.

7. I would be personally upset if someone I'd known for a long period of time revealed to me that they used to be another sex/gender.
8. In public, I avoid people whose gender is unclear to me.

Adapted from Stroumsa, D., Shires, D.A., Richardson, C.R., Jaffee, K.D. and Woodford, M.R. (2019), Transphobia rather than education predicts provider knowledge of transgender health care. Med Educ, 53: 398-407. https://doi.org/10.1111/medu.13796.

Demographics

- What is your gender identity?
    - Male
    - Female
    - Non-binary / genderqueer
    - Other
- Do you identify as transgender and/or non-binary/genderqueer?
    - Yes
    - No
    - Unsure
- Racial/ethnic/cultural background (Select all that apply)
    - American Indian or Alaska Native (For example: Aztec, Blackfeet Tribe, Mayan, Navajo Nation, Native Village of Barrow (Utqiagvik) Inupiat Traditional Government, Nome Eskimo Community, etc.)
    - Asian (For example: Asian Indian, Chinese, Filipino, Japanese, Korean, Vietnamese, etc.)
    - Black, African American, or African (For example: African American, Ethiopian, Haitian, Jamaican, Nigerian, Somali, etc.)
    - Hispanic, Latino, or Spanish (For example: Colombian, Cuban, Dominican, Mexican or Mexican American, Puerto Rican, Salvadoran, etc.)
    - Middle Eastern or North African (For example: Algerian, Egyptian, Iranian, Lebanese, Moroccan, Syrian, etc.)
    - Native Hawaiian or other Pacific Islander (For example: Chamorro, Fijian, Marshallese, Native Hawaiian, Tongan, etc.)
    - White (For example: English, European, French, German, Irish, Italian, Polish, etc.)
    - None of these fully describe me
    - Prefer not to answer
- Do you identify as part of the LGBTQIA+ community?
    - Yes
    - No
    - Prefer not to answer
- Does an immediate family member identify as part of the LGBTQIA+ community?

    - Yes
    - No
    - Prefer not to answer
- Does an extended family member or close friend identify as part of the LGBTQIA+ community?
    - Yes
    - No
    - Prefer not to answer
- ***Please create a unique identifier by using the last four digits of your phone number and the first letter of your last name (e.g. 1234A or 3452T)***

POST-SURVEY

- ***What are the last four digits of your phone number and the first letter of your last name (e.g. 1234A or 3452T)***

Self-Assessed Knowledge Scale

Please rate your knowledge on the following BEFORE completion of the training:

	**Very knowledgeable**	**Knowledgeable**	**Aware but do not know much about it**	**Not at all knowledgeable**
Definition of LGBTQIA+				
Difference between sex, gender identity, gender expression, and sexual orientation				
Use of LGBTQIA+-inclusive terminology in medical setting				
Current breast cancer guidelines & recommendations for LGBTQIA+ patients				
Ways to practice demonstrating support and a safe place for LGBTQIA+ patients				

Knowledge Survey

9. Out of the options provided, what best represents the definition of gender?
    - A person's internal self-understanding and self-identification
        - Correct choice
    - The way a person presents themselves to the world

- The expression of genetic information in the body
- A person's genetic makeup (XX, XY chromosomes)

10. Which of these describes a person with a non-binary gender identity?
    - Someone who is attracted to both men and women
    - Someone who is attracted to neither men or women
    - Someone who identifies as a woman but was called a boy when they were born
    - Someone who identifies as neither a man nor a woman
        - Correct choice

11. Which of these describes a trans man?
    - A man who is attracted to other men
    - A man who is attracted to men and women
    - A person who was designed male at birth but identifies as a woman
    - A person who was designated female at birth but identifies as a man
        - Correct choice
    - A person who was designated male at birth but identifies as a man sometimes and a woman other times
    - A person who was designated female at birth and identifies as neither a man nor a woman

12. Which of these are environmental features of healthcare spaces that can make a transgender or non-binary person feel welcome? (choose all that apply)
    a. Having separate male and female bathrooms
    b. Staff wearing pronoun pins/stickers
    c. Gender-neutral names for spaces (e.g. "Center for Reproductive Health" rather than "Center for Women's Health")
    d. Intake forms that allow someone to choose between "male" "female" and "other" for sex
    e. Intake forms that allow for someone to indicate their chosen or preferred name
        i. Correct answers b, c, e

13. Which would be the most inclusive way of calling someone named "John Smith" who was wearing a dress and had long hair from the waiting room?
    - (at door) "Mrs. Smith?" (behind door) "Hello, I'm Taylor and I use she/her pronouns. Let's go back to the room"
    - (at door) "Mr. Smith?" (behind door) "Hello, I'm Taylor. Let's go back to the room"
    - (at door) "Smith?" (behind door) "Hello, can you confirm your date of birth? I'm Taylor and I use she/her pronouns. Let's go back to the room"
        - Correct choice
    - (at door) "Smith?" (behind door) "Hello, I'm Taylor. Let's go back to the room"

14. You are the clinical service specialist and ask the partner of patient Jane (legal name John) Smith who uses she/her pronouns what their relationship to "him" is. The partner corrects you. What is the most appropriate next thing to say?
    - "I'm so sorry, I'm new to this. I'll do better next time."
    - "I'm sorry, it's so confusing when it says John on here. I'm trying to learn to get better at this."
    - "I'm sorry, what's your relationship to *her*? Are you her emergency contact? Can I have her insurance card?"
        - Correct choice

15. You are the clinical service specialist and a person with a beard comes to your station in the waiting room to check in for a mammogram. You ask for his name and he says "Matthew Jones". You do not see an appointment today for a Matthew Jones, but see one in a few minutes for a "Jane Jones". What would be the best response?
    a. "Are you transgender? I see a different name in here" Then update legal name in the computer.
    b. "Can you confirm your date of birth and address?" It if matches date of birth and address for Jane Jones, tell him that he is registered under Jane and will need to give that name next time to confirm identity.
    c. "Can you confirm your date of birth and address?" It if matches date of birth and address for Jane Jones, ask for an ID. If it says Jane, add "Matthew" as chosen name and/or tell staff so they know to call him Matthew.
        i. Correct choice
    d. "Could it be under Jane Jones?" Then update chosen name in the system to Matthew

16. What is an example of transparency in Trauma-Informed Care?
    a. Allowing patients to take breaks or stop an exam/procedure if needed
    b. Explaining the importance of each step of a procedure and what to expect
        i. Correct choice
    c. Only asking questions relevant to your role on the team (e.g. not asking about genital surgery if you do not need to know)
    d. Use proper draping techniques or ensuring privacy before asking sensitive questions

Gender Attitudes Scale

(all scored on a seven-point Likert scale from Strongly Agree to Strongly Disagree)

9. It is important for me to be able to identify someone as only a man or only a woman upon meeting them.

10. I believe that the male/female social dichotomy is natural.
11. I am not comfortable around people who do not conform to traditional gender roles, such as confident & authoritative women or emotion-driven men.
12. I believe that a person can never change their gender in their lifetime.
13. A person’s genitalia defines what gender they are, e.g., a penis defines a person as being a man, a vagina defines a person as being a woman.
14. I think there is something wrong with a person who says that they are neither a man nor a woman.
15. I would be personally upset if someone I’d known for a long period of time revealed to me that they used to be another sex/gender.
16. In public, I avoid people whose gender is unclear to me.

Adapted from Stroumsa, D., Shires, D.A., Richardson, C.R., Jaffee, K.D. and Woodford, M.R. (2019), Transphobia rather than education predicts provider knowledge of transgender health care. Med Educ, 53: 398-407. https://doi.org/10.1111/medu.13796.
