# Supplementary material for: Training imaging professionals on transgender-inclusive breast cancer screening practices
Source: Cancer Causes Control. 2026 Sep 18;37(10):163. doi: 10.1007/s10552-026-02241-1 (PMC13588859; doi:10.1007/s10552-026-02241-1)
Supplement: Supplementary file 2 [file 10552_2026_2241_MOESM2_ESM.pdf]

STATISTICAL OUTPUT

Tests of Pre to Post Differences in Knowledge

T-Test

Paired Samples Statistics

		Mean	N	Std. Deviation	Std. Error Mean
Pair 1	know1preR	2.6429	14	.84190	.22501
	know1postR	3.1429	14	.66299	.17719
Pair 2	know2preR	2.4286	14	.85163	.22761
	know2postR	3.1429	14	.77033	.20588
Pair 3	know3preR	2.2143	14	.89258	.23855
	know3postR	3.1429	14	.66299	.17719
Pair 4	know4preR	2.3571	14	1.00821	.26945
	know4postR	3.0714	14	.61573	.16456
Pair 5	know5preR	2.3571	14	.84190	.22501
	know5postR	3.1429	14	.53452	.14286
Pair 6	knowMeanpreR	2.4000	14	.75243	.20110
	knowMeanpostR	3.1286	14	.56354	.15061

Paired Samples Test

		Paired Differences							Significance	
					95% Confidence Interval of the Difference					
		Mean	Std. Deviation	Std. Error Mean	Lower	Upper	t	df	One-Sided p	Two-Sided p
Pair 1	know1preR - know1postR	-.50000	.51887	.13868	-.79959	-.20041	-3.606	13	.002	.003
Pair 2	know2preR - know2postR	-.71429	.72627	.19410	-1.13362	-.29495	-3.680	13	.001	.003
Pair 3	know3preR - know3postR	-.92857	.82874	.22149	-1.40707	-.45007	-4.192	13	<.001	.001
Pair 4	know4preR - know4postR	-.71429	.99449	.26579	-1.28849	-.14008	-2.687	13	.009	.019
Pair 5	know5preR - know5postR	-.78571	.89258	.23855	-1.30108	-.27035	-3.294	13	.003	.006
Pair 6	knowMeanpreR - knowMeanpostR	-.72857	.62071	.16589	-1.08696	-.37019	-4.392	13	<.001	<.001

Paired Samples Effect Sizes

			Standardizer[a]	Point Estimate	95% Confidence Interval	
					Lower	Upper
Pair 1	know1preR - know1postR	Cohen's d	.51887	-.964	-1.591	-.312
		Hedges' correction	.55142	-.907	-1.497	-.294
Pair 2	know2preR - know2postR	Cohen's d	.72627	-.983	-1.615	-.327
		Hedges' correction	.77182	-.925	-1.519	-.308
Pair 3	know3preR - know3postR	Cohen's d	.82874	-1.120	-1.783	-.432
		Hedges' correction	.88072	-1.054	-1.678	-.407
Pair 4	know4preR - know4postR	Cohen's d	.99449	-.718	-1.298	-.117
		Hedges' correction	1.05686	-.676	-1.222	-.110
Pair 5	know5preR - know5postR	Cohen's d	.89258	-.880	-1.490	-.247
		Hedges' correction	.94856	-.828	-1.402	-.232
Pair 6	knowMeanpreR - knowMeanpostR	Cohen's d	.62071	-1.174	-1.849	-.473
		Hedges' correction	.65964	-1.105	-1.740	-.445

a. The denominator used in estimating the effect sizes.
Cohen's d uses the sample standard deviation of the mean difference.
Hedges' correction uses the sample standard deviation of the mean difference, plus a correction factor.

Tests of Pre to Post Changes in GAS and Number of Correct Responses

T-Test

Paired Samples Statistics

		Mean	N	Std. Deviation	Std. Error Mean
Pair 1	GAStotpre	42.36	11	9.146	2.758
	GAStotpost	44.36	11	10.250	3.090
Pair 2	quiztotpre	8.08	12	1.730	.499
	quiztotpost	9.17	12	2.125	.613

Paired Samples Correlations

		N	Correlation	Significance	
				One-Sided p	Two-Sided p
Pair 1	GAStotpre & GAStotpost	11	.926	<.001	<.001
Pair 2	quiztotpre & quiztotpost	12	-.350	.132	.264

Paired Samples Test

		Paired Differences					t	df	Significance	
					95% Confidence Interval of the Difference					
		Mean	Std. Deviation	Std. Error Mean	Lower	Upper			One-Sided p	Two-Sided p
Pair 1	GAStotpre - GAStotpost	-2.000	3.873	1.168	-4.602	.602	-1.713	10	.059	.118
Pair 2	quiztotpre - quiztotpost	-1.083	3.175	.917	-3.101	.934	-1.182	11	.131	.262

Paired Samples Effect Sizes

			Standardizer[a]	Point Estimate	95% Confidence Interval	
					Lower	Upper
Pair 1	GAStotpre - GAStotpost	Cohen's d	3.873	-.516	-1.137	.126
		Hedges' correction	4.197	-.477	-1.049	.117
Pair 2	quiztotpre - quiztotpost	Cohen's d	3.175	-.341	-.917	.249
		Hedges' correction	3.415	-.317	-.853	.232

a. The denominator used in estimating the effect sizes.
Cohen's d uses the sample standard deviation of the mean difference.
Hedges' correction uses the sample standard deviation of the mean difference, plus a correction factor.
